# Supplementary material for: Mechanistic insight into the role of AUXIN RESISTANCE4 in trafficking of AUXIN1 and LIKE AUX1-2
Source: Plant Physiol. 2023 Sep 30;194(1):422–33. doi: 10.1093/plphys/kiad506 (PMC10756756; doi:10.1093/plphys/kiad506)
Supplement: kiad506_Supplementary_Data [file kiad506_supplementary_data.zip › Suppemental Dataset S1/5445e4f6eaa2385e-AtAXR4/hit_summary.html]

Phyre 2 Results for AtAXR4\_\_\_

|  |  |
| --- | --- |
| Email | charlie.hodgman@nottinghamac.uk |
| Description | AtAXR4\_\_\_ |
| Date | Tue Jan 10 14:09:39 GMT 2023 || Unique Job ID | 5445e4f6eaa2385e |

|  |  |
| --- | --- |
| Detailed template information |  |

| # | Template | Alignment Coverage | 3D Model | Confidence | % i.d. | Template Information |
| --- | --- | --- | --- | --- | --- | --- |

|  |  |  |  |  |  |  |
| --- | --- | --- | --- | --- | --- | --- |
| 1 | c6g75B\_ | --- \| --- \| --- |  | 100.0 | 24 | **PDB header:**hydrolase **Chain:** B: **PDB Molecule:**common ancestor of haloalkane dehalogenase and renilla  **PDBTitle:** crystal structure of the common ancestor of haloalkane dehalogenases2 and renilla luciferase (anchld-rluc)  **PDB Entry:** PDBe RCSB PDBj |
| 2 | c2vf2A\_ | --- \| --- \| --- |  | 100.0 | 24 | **PDB header:**hydrolase **Chain:** A: **PDB Molecule:**2-hydroxy-6-oxo-6-phenylhexa-2,4-dienoate  **PDBTitle:** x-ray crystal structure of hsad from mycobacterium2 tuberculosis  **PDB Entry:** PDBe RCSB PDBj |
| 3 | c4mj3B\_ | --- \| --- \| --- |  | 100.0 | 22 | **PDB header:**hydrolase **Chain:** B: **PDB Molecule:**haloalkane dehalogenase;  **PDBTitle:** haloalkane dehalogenase dmra from mycobacterium rhodesiae js60  **PDB Entry:** PDBe RCSB PDBj |
| 4 | d1bn7a\_ | --- \| --- \| --- |  | 100.0 | 23 | **Fold:** alpha/beta-Hydrolases **Superfamily:** alpha/beta-Hydrolases **Family:** Haloalkane dehalogenase **PDB entry:** PDBe RCSB PDBj |
| 5 | c3i28A\_ | --- \| --- \| --- |  | 100.0 | 20 | **PDB header:**hydrolase **Chain:** A: **PDB Molecule:**epoxide hydrolase 2;  **PDBTitle:** crystal structure of soluble epoxide hydrolase  **PDB Entry:** PDBe RCSB PDBj |
| 6 | c1cr6A\_ | --- \| --- \| --- |  | 100.0 | 20 | **PDB header:**hydrolase **Chain:** A: **PDB Molecule:**epoxide hydrolase;  **PDBTitle:** crystal structure of murine soluble epoxide hydrolase2 complexed with cpu inhibitor  **PDB Entry:** PDBe RCSB PDBj |
| 7 | c6y9gB\_ | --- \| --- \| --- |  | 100.0 | 22 | **PDB header:**hydrolase **Chain:** B: **PDB Molecule:**ancestral haloalkane dehalogenase anchld5;  **PDBTitle:** crystal structure of putative ancestral haloalkane dehalogenase2 anchld5 (node 5)  **PDB Entry:** PDBe RCSB PDBj |
| 8 | c4ns4A\_ | --- \| --- \| --- |  | 100.0 | 21 | **PDB header:**hydrolase **Chain:** A: **PDB Molecule:**alpha/beta hydrolase fold protein;  **PDBTitle:** crystal structure of cold-active estarase from psychrobacter2 cryohalolentis k5t  **PDB Entry:** PDBe RCSB PDBj |
| 9 | d1mj5a\_ | --- \| --- \| --- |  | 100.0 | 20 | **Fold:** alpha/beta-Hydrolases **Superfamily:** alpha/beta-Hydrolases **Family:** Haloalkane dehalogenase **PDB entry:** PDBe RCSB PDBj |
| 10 | c3u1tA\_ | --- \| --- \| --- |  | 100.0 | 21 | **PDB header:**hydrolase **Chain:** A: **PDB Molecule:**dmma haloalkane dehalogenase;  **PDBTitle:** haloalkane dehalogenase, dmma, of marine microbial origin  **PDB Entry:** PDBe RCSB PDBj |
| 11 | c4c6hA\_ | --- \| --- \| --- |  | 100.0 | 21 | **PDB header:**hydrolase **Chain:** A: **PDB Molecule:**haloalkane dehalogenase;  **PDBTitle:** haloalkane dehalogenase with 1-hexanol  **PDB Entry:** PDBe RCSB PDBj |
| 12 | c3a2nF\_ | --- \| --- \| --- |  | 100.0 | 20 | **PDB header:**hydrolase **Chain:** F: **PDB Molecule:**haloalkane dehalogenase;  **PDBTitle:** crystal structure of dbja (wild type type ii p21)  **PDB Entry:** PDBe RCSB PDBj |
| 13 | c3wibB\_ | --- \| --- \| --- |  | 100.0 | 20 | **PDB header:**hydrolase **Chain:** B: **PDB Molecule:**haloalkane dehalogenase;  **PDBTitle:** crystal structure of y109w mutant haloalkane dehalogenase data from2 agrobacterium tumefaciens c58  **PDB Entry:** PDBe RCSB PDBj |
| 14 | c7jqzE\_ | --- \| --- \| --- |  | 100.0 | 21 | **PDB header:**hydrolase **Chain:** E: **PDB Molecule:**alpha/beta hydrolase fold;  **PDBTitle:** crystal structure of cfl2 wild-type from burkholderia cenocepacia  **PDB Entry:** PDBe RCSB PDBj |
| 15 | d1cr6a2 | --- \| --- \| --- |  | 100.0 | 20 | **Fold:** alpha/beta-Hydrolases **Superfamily:** alpha/beta-Hydrolases **Family:** Epoxide hydrolase **PDB entry:** PDBe RCSB PDBj |
| 16 | d1zd3a2 | --- \| --- \| --- |  | 100.0 | 20 | **Fold:** alpha/beta-Hydrolases **Superfamily:** alpha/beta-Hydrolases **Family:** Epoxide hydrolase **PDB entry:** PDBe RCSB PDBj |
| 17 | c2e3jA\_ | --- \| --- \| --- |  | 100.0 | 20 | **PDB header:**hydrolase **Chain:** A: **PDB Molecule:**epoxide hydrolase ephb;  **PDBTitle:** the crystal structure of epoxide hydrolase b (rv1938) from2 mycobacterium tuberculosis at 2.1 angstrom  **PDB Entry:** PDBe RCSB PDBj |
| 18 | c7avrG\_ | --- \| --- \| --- |  | 100.0 | 18 | **PDB header:**hydrolase **Chain:** G: **PDB Molecule:**haloalkane dehalogenase 1;  **PDBTitle:** the tetrameric structure of haloalkane dehalogenase dpaa from2 paraglaciecola agarilytica no2  **PDB Entry:** PDBe RCSB PDBj |
| 19 | c2qvbA\_ | --- \| --- \| --- |  | 100.0 | 20 | **PDB header:**hydrolase **Chain:** A: **PDB Molecule:**haloalkane dehalogenase 3;  **PDBTitle:** crystal structure of haloalkane dehalogenase rv2579 from mycobacterium2 tuberculosis  **PDB Entry:** PDBe RCSB PDBj |
| 20 | c5xmdA\_ | --- \| --- \| --- |  | 100.0 | 19 | **PDB header:**hydrolase **Chain:** A: **PDB Molecule:**epoxide hydrolase a;  **PDBTitle:** crystal structure of epoxide hydrolase vreh1 from vigna radiata  **PDB Entry:** PDBe RCSB PDBj |
| 21 | d1hkha\_ | --- \| --- \| --- | not modelled | 100.0 | 20 | **Fold:** alpha/beta-Hydrolases **Superfamily:** alpha/beta-Hydrolases **Family:** Haloperoxidase **PDB entry:** PDBe RCSB PDBj |
| 22 | c2pseA\_ | --- \| --- \| --- | not modelled | 100.0 | 18 | **PDB header:**oxidoreductase **Chain:** A: **PDB Molecule:**renilla-luciferin 2-monooxygenase;  **PDBTitle:** crystal structures of the luciferase and green fluorescent protein2 from renilla reniformis  **PDB Entry:** PDBe RCSB PDBj |
| 23 | c5ng7B\_ | --- \| --- \| --- | not modelled | 100.0 | 20 | **PDB header:**hydrolase **Chain:** B: **PDB Molecule:**epoxide hydrolase;  **PDBTitle:** novel epoxide hydrolases belonging to the alpha/beta hydrolases2 superfamily in metagenomes from hot environments  **PDB Entry:** PDBe RCSB PDBj |
| 24 | d2rhwa1 | --- \| --- \| --- | not modelled | 100.0 | 19 | **Fold:** alpha/beta-Hydrolases **Superfamily:** alpha/beta-Hydrolases **Family:** Carbon-carbon bond hydrolase **PDB entry:** PDBe RCSB PDBj |
| 25 | c4psuA\_ | --- \| --- \| --- | not modelled | 100.0 | 19 | **PDB header:**hydrolase **Chain:** A: **PDB Molecule:**alpha/beta hydrolase;  **PDBTitle:** crystal structure of alpha/beta hydrolase from rhodopseudomonas2 palustris cga009  **PDB Entry:** PDBe RCSB PDBj |
| 26 | d1a8qa\_ | --- \| --- \| --- | not modelled | 100.0 | 20 | **Fold:** alpha/beta-Hydrolases **Superfamily:** alpha/beta-Hydrolases **Family:** Haloperoxidase **PDB entry:** PDBe RCSB PDBj |
| 27 | c6f9oA\_ | --- \| --- \| --- | not modelled | 100.0 | 18 | **PDB header:**hydrolase **Chain:** A: **PDB Molecule:**haloalkane dehalogenase;  **PDBTitle:** crystal structure of cold-adapted haloalkane dehalogenase dpca from2 psychrobacter cryohalolentis k5  **PDB Entry:** PDBe RCSB PDBj |
| 28 | c2wirB\_ | --- \| --- \| --- | not modelled | 98.8 | 20 | **PDB header:**hydrolase **Chain:** B: **PDB Molecule:**alpha/beta hydrolase fold-3 domain protein;  **PDBTitle:** hyperthermophilic esterase from the archeon pyrobaculum2 calidifontis  **PDB Entry:** PDBe RCSB PDBj |
| 29 | c7wwfD\_ | --- \| --- \| --- | not modelled | 100.0 | 19 | **PDB header:**biosynthetic protein **Chain:** D: **PDB Molecule:**esterase;  **PDBTitle:** crystal structure of bioh3 from mycolicibacterium smegmatis  **PDB Entry:** PDBe RCSB PDBj |
| 30 | c5frdA\_ | --- \| --- \| --- | not modelled | 100.0 | 20 | **PDB header:**hydrolase **Chain:** A: **PDB Molecule:**carboxylesterase (est-2);  **PDBTitle:** structure of a thermophilic esterase  **PDB Entry:** PDBe RCSB PDBj |
| 31 | c6i8wB\_ | --- \| --- \| --- | not modelled | 100.0 | 16 | **PDB header:**hydrolase **Chain:** B: **PDB Molecule:**alpha/beta fold hydrolase;  **PDBTitle:** crystal structure of a membrane phospholipase a, a novel bacterial2 virulence factor  **PDB Entry:** PDBe RCSB PDBj |
| 32 | c4opmB\_ | --- \| --- \| --- | not modelled | 100.0 | 17 | **PDB header:**hydrolase **Chain:** B: **PDB Molecule:**lipase;  **PDBTitle:** crystal structure of a putative lipase (lip1) from acinetobacter2 baumannii aye at 1.70 a resolution  **PDB Entry:** PDBe RCSB PDBj |
| 33 | c4inzB\_ | --- \| --- \| --- | not modelled | 100.0 | 19 | **PDB header:**hydrolase **Chain:** B: **PDB Molecule:**soluble epoxide hydrolase;  **PDBTitle:** the crystal structure of m145a mutant of an epoxide hydrolase from2 bacillus megaterium  **PDB Entry:** PDBe RCSB PDBj |
| 34 | c3qitB\_ | --- \| --- \| --- | not modelled | 100.0 | 19 | **PDB header:**hydrolase **Chain:** B: **PDB Molecule:**polyketide synthase;  **PDBTitle:** thioesterase domain from curacin biosynthetic pathway  **PDB Entry:** PDBe RCSB PDBj |
| 35 | c2xt0A\_ | --- \| --- \| --- | not modelled | 100.0 | 18 | **PDB header:**hydrolase **Chain:** A: **PDB Molecule:**haloalkane dehalogenase;  **PDBTitle:** dehalogenase dppa from plesiocystis pacifica sir-i  **PDB Entry:** PDBe RCSB PDBj |
| 36 | c5w8pA\_ | --- \| --- \| --- | not modelled | 100.0 | 16 | **PDB header:**transferase **Chain:** A: **PDB Molecule:**homoserine o-acetyltransferase;  **PDBTitle:** homoserine transacetylase metx from mycobacterium abscessus  **PDB Entry:** PDBe RCSB PDBj |
| 37 | c1u2eA\_ | --- \| --- \| --- | not modelled | 100.0 | 18 | **PDB header:**hydrolase **Chain:** A: **PDB Molecule:**2-hydroxy-6-ketonona-2,4-dienedioic acid  **PDBTitle:** crystal structure of the c-c bond hydrolase mhpc  **PDB Entry:** PDBe RCSB PDBj |
| 38 | c6kxhD\_ | --- \| --- \| --- | not modelled | 100.0 | 19 | **PDB header:**hydrolase **Chain:** D: **PDB Molecule:**putative hydrolase;  **PDBTitle:** alp1u\_y247f mutant in complex with fluostatin c  **PDB Entry:** PDBe RCSB PDBj |
| 39 | c6unwA\_ | --- \| --- \| --- | not modelled | 100.0 | 18 | **PDB header:**hydrolase **Chain:** A: **PDB Molecule:**soluble epoxide hydrolase;  **PDBTitle:** epoxide hydrolase from an endophytic streptomyces  **PDB Entry:** PDBe RCSB PDBj |
| 40 | c5xb6D\_ | --- \| --- \| --- | not modelled | 99.9 | 18 | **PDB header:**hydrolase **Chain:** D: **PDB Molecule:**uncharacterized protein ycjy;  **PDBTitle:** crystal structure of ycjy from e. coli  **PDB Entry:** PDBe RCSB PDBj |
| 41 | c5cw2C\_ | --- \| --- \| --- | not modelled | 100.0 | 18 | **PDB header:**hydrolase **Chain:** C: **PDB Molecule:**putative epoxide hydrolase epha;  **PDBTitle:** crystal structure of epoxide hydrolase a from mycobacterium2 thermoresistibile  **PDB Entry:** PDBe RCSB PDBj |
| 42 | d2hu7a2 | --- \| --- \| --- | not modelled | 99.6 | 19 | **Fold:** alpha/beta-Hydrolases **Superfamily:** alpha/beta-Hydrolases **Family:** Acylamino-acid-releasing enzyme, C-terminal donain **PDB entry:** PDBe RCSB PDBj |
| 43 | c4b9aA\_ | --- \| --- \| --- | not modelled | 100.0 | 17 | **PDB header:**hydrolase **Chain:** A: **PDB Molecule:**probable epoxide hydrolase;  **PDBTitle:** structure of a putative epoxide hydrolase from pseudomonas2 aeruginosa.  **PDB Entry:** PDBe RCSB PDBj |
| 44 | c8e18A\_ | --- \| --- \| --- | not modelled | 99.9 | 15 | **PDB header:**hydrolase **Chain:** A: **PDB Molecule:**secreted hydrolase;  **PDBTitle:** crystal structure of apo tnmk1  **PDB Entry:** PDBe RCSB PDBj |
| 45 | c5jrlC\_ | --- \| --- \| --- | not modelled | 99.3 | 20 | **PDB header:**hydrolase **Chain:** C: **PDB Molecule:**dipeptidyl aminopeptidases/acylaminoacyl-peptidases-like  **PDBTitle:** crystal structure of the sphingopyxin i lasso peptide isopeptidase2 spi-isop (native)  **PDB Entry:** PDBe RCSB PDBj |
| 46 | c4zxiA\_ | --- \| --- \| --- | not modelled | 99.4 | 16 | **PDB header:**biosynthetic protein **Chain:** A: **PDB Molecule:**tyrocidine synthetase 3;  **PDBTitle:** crystal structure of holo-ab3403 a four domain nonribosomal peptide2 synthetase bound to amp and glycine  **PDB Entry:** PDBe RCSB PDBj |
| 47 | d1brta\_ | --- \| --- \| --- | not modelled | 100.0 | 18 | **Fold:** alpha/beta-Hydrolases **Superfamily:** alpha/beta-Hydrolases **Family:** Haloperoxidase **PDB entry:** PDBe RCSB PDBj |
| 48 | d1fj2a\_ | --- \| --- \| --- | not modelled | 99.8 | 22 | **Fold:** alpha/beta-Hydrolases **Superfamily:** alpha/beta-Hydrolases **Family:** Carboxylesterase/thioesterase 1 **PDB entry:** PDBe RCSB PDBj |
| 49 | c4uhhA\_ | --- \| --- \| --- | not modelled | 100.0 | 17 | **PDB header:**hydrolase **Chain:** A: **PDB Molecule:**esterase;  **PDBTitle:** structural studies of a thermophilic esterase from2 thermogutta terrifontis (cacodylate complex)  **PDB Entry:** PDBe RCSB PDBj |
| 50 | d1qo7a\_ | --- \| --- \| --- | not modelled | 100.0 | 18 | **Fold:** alpha/beta-Hydrolases **Superfamily:** alpha/beta-Hydrolases **Family:** Epoxide hydrolase **PDB entry:** PDBe RCSB PDBj |
| 51 | c3kdaB\_ | --- \| --- \| --- | not modelled | 100.0 | 18 | **PDB header:**hydrolase **Chain:** B: **PDB Molecule:**cftr inhibitory factor (cif);  **PDBTitle:** crystal structure of the cftr inhibitory factor cif with the h269a2 mutation  **PDB Entry:** PDBe RCSB PDBj |
| 52 | c4rncB\_ | --- \| --- \| --- | not modelled | 100.0 | 18 | **PDB header:**hydrolase **Chain:** B: **PDB Molecule:**esterase;  **PDBTitle:** crystal structure of an esterase rhest1 from rhodococcus sp. ecu1013  **PDB Entry:** PDBe RCSB PDBj |
| 53 | c6u2mC\_ | --- \| --- \| --- | not modelled | 100.0 | 22 | **PDB header:**hydrolase **Chain:** C: **PDB Molecule:**halocamp v2;  **PDBTitle:** crystal structure of a halotag-based calcium indicator, halocamp v2,2 bound to jf635  **PDB Entry:** PDBe RCSB PDBj |
| 54 | c4d9jI\_ | --- \| --- \| --- | not modelled | 100.0 | 16 | **PDB header:**de novo protein **Chain:** I: **PDB Molecule:**designed 16nm tetrahedral protein cage containing non-haem  **PDBTitle:** structure of a 16 nm protein cage designed by fusing symmetric2 oligomeric domains  **PDB Entry:** PDBe RCSB PDBj |
| 55 | c5uohA\_ | --- \| --- \| --- | not modelled | 99.9 | 15 | **PDB header:**hydrolase **Chain:** A: **PDB Molecule:**carboxylesterase a;  **PDBTitle:** crystal structure of hip1 (rv2224c) t466a mutant  **PDB Entry:** PDBe RCSB PDBj |
| 56 | d1f0na\_ | --- \| --- \| --- | not modelled | 99.5 | 18 | **Fold:** alpha/beta-Hydrolases **Superfamily:** alpha/beta-Hydrolases **Family:** Mycobacterial antigens **PDB entry:** PDBe RCSB PDBj |
| 57 | c6aaeA\_ | --- \| --- \| --- | not modelled | 98.9 | 19 | **PDB header:**hydrolase **Chain:** A: **PDB Molecule:**esterase;  **PDBTitle:** crystal structure of chloramphenicol-metabolizaing enzyme estdl136  **PDB Entry:** PDBe RCSB PDBj |
| 58 | c5hk8A\_ | --- \| --- \| --- | not modelled | 100.0 | 19 | **PDB header:**hydrolase **Chain:** A: **PDB Molecule:**probable pheophorbidase;  **PDBTitle:** crystal structure of a methylesterase protein mes16 from arabidopsis  **PDB Entry:** PDBe RCSB PDBj |
| 59 | c6ojdB\_ | --- \| --- \| --- | not modelled | 100.0 | 20 | **PDB header:**hydrolase **Chain:** B: **PDB Molecule:**nocb;  **PDBTitle:** a high-resolution crystal structure of covalent complex of nocb2 thioesterase domain with fluorophosphonate nocardicin g analog  **PDB Entry:** PDBe RCSB PDBj |
| 60 | c4lxgA\_ | --- \| --- \| --- | not modelled | 100.0 | 17 | **PDB header:**hydrolase **Chain:** A: **PDB Molecule:**mcp hydrolase;  **PDBTitle:** crystal structure of dxnb2, a carbon - carbon bond hydrolase from2 sphingomonas wittichii rw1  **PDB Entry:** PDBe RCSB PDBj |
| 61 | c5yhpB\_ | --- \| --- \| --- | not modelled | 100.0 | 17 | **PDB header:**hydrolase **Chain:** B: **PDB Molecule:**cold active proline iminopeptidase;  **PDBTitle:** proline iminopeptidase from psychrophilic yeast glaciozyma antarctica  **PDB Entry:** PDBe RCSB PDBj |
| 62 | c3u0vA\_ | --- \| --- \| --- | not modelled | 99.6 | 21 | **PDB header:**hydrolase **Chain:** A: **PDB Molecule:**lysophospholipase-like protein 1;  **PDBTitle:** crystal structure analysis of human lyplal1  **PDB Entry:** PDBe RCSB PDBj |
| 63 | d1b6ga\_ | --- \| --- \| --- | not modelled | 100.0 | 15 | **Fold:** alpha/beta-Hydrolases **Superfamily:** alpha/beta-Hydrolases **Family:** Haloalkane dehalogenase **PDB entry:** PDBe RCSB PDBj |
| 64 | c1y37A\_ | --- \| --- \| --- | not modelled | 100.0 | 16 | **PDB header:**hydrolase **Chain:** A: **PDB Molecule:**fluoroacetate dehalogenase;  **PDBTitle:** structure of fluoroacetate dehalogenase from burkholderia sp. fa1  **PDB Entry:** PDBe RCSB PDBj |
| 65 | c7dq9A\_ | --- \| --- \| --- | not modelled | 100.0 | 17 | **PDB header:**hydrolase **Chain:** A: **PDB Molecule:**predicted hydrolases or acyltransferases (alpha/beta  **PDBTitle:** crystal structure of a type-a feruloyl esterase from gut alistipes2 shahii  **PDB Entry:** PDBe RCSB PDBj |
| 66 | d1ivya\_ | --- \| --- \| --- | not modelled | 99.0 | 17 | **Fold:** alpha/beta-Hydrolases **Superfamily:** alpha/beta-Hydrolases **Family:** Serine carboxypeptidase-like **PDB entry:** PDBe RCSB PDBj |
| 67 | c2zyiB\_ | --- \| --- \| --- | not modelled | 99.8 | 19 | **PDB header:**hydrolase **Chain:** B: **PDB Molecule:**lipase, putative;  **PDBTitle:** a. fulgidus lipase with fatty acid fragment and calcium  **PDB Entry:** PDBe RCSB PDBj |
| 68 | c5mxpB\_ | --- \| --- \| --- | not modelled | 100.0 | 17 | **PDB header:**hydrolase **Chain:** B: **PDB Molecule:**alpha/beta hydrolase;  **PDBTitle:** haloalkane dehalogenase dmxa from marinobacter sp. elb17 possessing a2 unique catalytic residue  **PDB Entry:** PDBe RCSB PDBj |
| 69 | c3r3xA\_ | --- \| --- \| --- | not modelled | 100.0 | 16 | **PDB header:**hydrolase **Chain:** A: **PDB Molecule:**fluoroacetate dehalogenase;  **PDBTitle:** crystal structure of the fluoroacetate dehalogenase rpa1163 -2 asp110asn/bromoacetate  **PDB Entry:** PDBe RCSB PDBj |
| 70 | c2hdwB\_ | --- \| --- \| --- | not modelled | 99.9 | 16 | **PDB header:**hydrolase **Chain:** B: **PDB Molecule:**hypothetical protein pa2218;  **PDBTitle:** crystal structure of hypothetical protein pa2218 from pseudomonas2 aeruginosa  **PDB Entry:** PDBe RCSB PDBj |
| 71 | d1wpxa1 | --- \| --- \| --- | not modelled | 98.8 | 15 | **Fold:** alpha/beta-Hydrolases **Superfamily:** alpha/beta-Hydrolases **Family:** Serine carboxypeptidase-like **PDB entry:** PDBe RCSB PDBj |
| 72 | c7c4dA\_ | --- \| --- \| --- | not modelled | 100.0 | 17 | **PDB header:**hydrolase **Chain:** A: **PDB Molecule:**putative esterase;  **PDBTitle:** marine microorganism esterase  **PDB Entry:** PDBe RCSB PDBj |
| 73 | c2y6vB\_ | --- \| --- \| --- | not modelled | 100.0 | 15 | **PDB header:**hydrolase **Chain:** B: **PDB Molecule:**peroxisomal membrane protein lpx1;  **PDBTitle:** peroxisomal alpha-beta-hydrolase lpx1 (yor084w) from saccharomyces2 cerevisiae (crystal form i)  **PDB Entry:** PDBe RCSB PDBj |
| 74 | c3i1iA\_ | --- \| --- \| --- | not modelled | 100.0 | 14 | **PDB header:**transferase **Chain:** A: **PDB Molecule:**homoserine o-acetyltransferase;  **PDBTitle:** x-ray crystal structure of homoserine o-acetyltransferase from2 bacillus anthracis  **PDB Entry:** PDBe RCSB PDBj |
| 75 | d1uk8a\_ | --- \| --- \| --- | not modelled | 100.0 | 16 | **Fold:** alpha/beta-Hydrolases **Superfamily:** alpha/beta-Hydrolases **Family:** Carbon-carbon bond hydrolase **PDB entry:** PDBe RCSB PDBj |
| 76 | d1e89a\_ | --- \| --- \| --- | not modelled | 100.0 | 17 | **Fold:** alpha/beta-Hydrolases **Superfamily:** alpha/beta-Hydrolases **Family:** Hydroxynitrile lyase-like **PDB entry:** PDBe RCSB PDBj |
| 77 | c5nfqA\_ | --- \| --- \| --- | not modelled | 100.0 | 16 | **PDB header:**hydrolase **Chain:** A: **PDB Molecule:**epoxide hydrolase belonging to alpha/beta hydrolase  **PDBTitle:** novel epoxide hydrolases belonging to the alpha/beta hydrolases2 superfamily in metagenomes from hot environments  **PDB Entry:** PDBe RCSB PDBj |
| 78 | c3kxpD\_ | --- \| --- \| --- | not modelled | 100.0 | 16 | **PDB header:**hydrolase **Chain:** D: **PDB Molecule:**alpha-(n-acetylaminomethylene)succinic acid hydrolase;  **PDBTitle:** crystal structure of e-2-(acetamidomethylene)succinate hydrolase  **PDB Entry:** PDBe RCSB PDBj |
| 79 | c6khlB\_ | --- \| --- \| --- | not modelled | 100.0 | 16 | **PDB header:**hydrolase **Chain:** B: **PDB Molecule:**hydrolase, alpha/beta domain protein;  **PDBTitle:** lipase (blocked form)  **PDB Entry:** PDBe RCSB PDBj |
| 80 | c1mo2A\_ | --- \| --- \| --- | not modelled | 100.0 | 20 | **PDB header:**transferase **Chain:** A: **PDB Molecule:**erythronolide synthase, modules 5 and 6;  **PDBTitle:** thioesterase domain from 6-deoxyerythronolide synthase (debs te), ph2 8.5  **PDB Entry:** PDBe RCSB PDBj |
| 81 | d1mo2a\_ | --- \| --- \| --- | not modelled | 100.0 | 20 | **Fold:** alpha/beta-Hydrolases **Superfamily:** alpha/beta-Hydrolases **Family:** Thioesterase domain of polypeptide, polyketide and fatty acid synthases **PDB entry:** PDBe RCSB PDBj |
| 82 | c2o2gA\_ | --- \| --- \| --- | not modelled | 99.8 | 21 | **PDB header:**hydrolase **Chain:** A: **PDB Molecule:**dienelactone hydrolase;  **PDBTitle:** crystal structure of dienelactone hydrolase (yp\_324580.1) from2 anabaena variabilis atcc 29413 at 1.92 a resolution  **PDB Entry:** PDBe RCSB PDBj |
| 83 | c7wwhB\_ | --- \| --- \| --- | not modelled | 99.9 | 18 | **PDB header:**hydrolase **Chain:** B: **PDB Molecule:**alpha/beta hydrolase;  **PDBTitle:** crystal structure of the geobacillus thermoglucosidasius feruloyl2 esterase gthfae  **PDB Entry:** PDBe RCSB PDBj |
| 84 | c3wwoA\_ | --- \| --- \| --- | not modelled | 100.0 | 17 | **PDB header:**lyase **Chain:** A: **PDB Molecule:**(s)-hydroxynitrile lyase;  **PDBTitle:** s-selective hydroxynitrile lyase from baliospermum montanum (apo1)  **PDB Entry:** PDBe RCSB PDBj |
| 85 | c2hu7A\_ | --- \| --- \| --- | not modelled | 99.9 | 17 | **PDB header:**hydrolase **Chain:** A: **PDB Molecule:**acylamino-acid-releasing enzyme;  **PDBTitle:** binding of inhibitors by acylaminoacyl peptidase  **PDB Entry:** PDBe RCSB PDBj |
| 86 | d1q0ra\_ | --- \| --- \| --- | not modelled | 100.0 | 16 | **Fold:** alpha/beta-Hydrolases **Superfamily:** alpha/beta-Hydrolases **Family:** Aclacinomycin methylesterase RdmC **PDB entry:** PDBe RCSB PDBj |
| 87 | d1hlga\_ | --- \| --- \| --- | not modelled | 100.0 | 14 | **Fold:** alpha/beta-Hydrolases **Superfamily:** alpha/beta-Hydrolases **Family:** Gastric lipase **PDB entry:** PDBe RCSB PDBj |
| 88 | c7qjmA\_ | --- \| --- \| --- | not modelled | 100.0 | 15 | **PDB header:**hydrolase **Chain:** A: **PDB Molecule:**alpha/beta-hydrolase (202);  **PDBTitle:** crystal structure of an alpha/beta-hydrolase enzyme from chloroflexus2 sp. ms-g (202)  **PDB Entry:** PDBe RCSB PDBj |
| 89 | c5esrA\_ | --- \| --- \| --- | not modelled | 100.0 | 15 | **PDB header:**hydrolase **Chain:** A: **PDB Molecule:**haloalkane dehalogenase;  **PDBTitle:** crystal structure of haloalkane dehalogenase (dcca) from caulobacter2 crescentus  **PDB Entry:** PDBe RCSB PDBj |
| 90 | d1j1ia\_ | --- \| --- \| --- | not modelled | 100.0 | 17 | **Fold:** alpha/beta-Hydrolases **Superfamily:** alpha/beta-Hydrolases **Family:** Carbon-carbon bond hydrolase **PDB entry:** PDBe RCSB PDBj |
| 91 | c1j1iA\_ | --- \| --- \| --- | not modelled | 100.0 | 17 | **PDB header:**hydrolase **Chain:** A: **PDB Molecule:**meta cleavage compound hydrolase;  **PDBTitle:** crystal structure of a his-tagged serine hydrolase involved2 in the carbazole degradation (carc enzyme)  **PDB Entry:** PDBe RCSB PDBj |
| 92 | c2vsqA\_ | --- \| --- \| --- | not modelled | 99.0 | 14 | **PDB header:**ligase **Chain:** A: **PDB Molecule:**surfactin synthetase subunit 3;  **PDBTitle:** structure of surfactin a synthetase c (srfa-c), a nonribosomal peptide2 synthetase termination module  **PDB Entry:** PDBe RCSB PDBj |
| 93 | c6ix4A\_ | --- \| --- \| --- | not modelled | 100.0 | 16 | **PDB header:**hydrolase **Chain:** A: **PDB Molecule:**microsomal epoxide hydrolase;  **PDBTitle:** structure of an epoxide hydrolase from aspergillus usamii e001 (aueh2)2 at 1.51 angstroms resolution  **PDB Entry:** PDBe RCSB PDBj |
| 94 | c2qmqA\_ | --- \| --- \| --- | not modelled | 100.0 | 16 | **PDB header:**signaling protein **Chain:** A: **PDB Molecule:**protein ndrg2;  **PDBTitle:** crystal structure of a n-myc downstream regulated 2 protein (ndrg2,2 syld, ndr2, ai182517, au040374) from mus musculus at 1.70 a3 resolution  **PDB Entry:** PDBe RCSB PDBj |
| 95 | d1a88a\_ | --- \| --- \| --- | not modelled | 100.0 | 16 | **Fold:** alpha/beta-Hydrolases **Superfamily:** alpha/beta-Hydrolases **Family:** Haloperoxidase **PDB entry:** PDBe RCSB PDBj |
| 96 | d2h7xa1 | --- \| --- \| --- | not modelled | 100.0 | 19 | **Fold:** alpha/beta-Hydrolases **Superfamily:** alpha/beta-Hydrolases **Family:** Thioesterase domain of polypeptide, polyketide and fatty acid synthases **PDB entry:** PDBe RCSB PDBj |
| 97 | c4qlaA\_ | --- \| --- \| --- | not modelled | 100.0 | 16 | **PDB header:**hydrolase **Chain:** A: **PDB Molecule:**juvenile hormone epoxide hydrolase;  **PDBTitle:** crystal structure of juvenile hormone epoxide hydrolase from the2 silkworm bombyx mori  **PDB Entry:** PDBe RCSB PDBj |
| 98 | c6ra2E\_ | --- \| --- \| --- | not modelled | 100.0 | 16 | **PDB header:**hydrolase **Chain:** E: **PDB Molecule:**putative dioxygenase (1h-3-hydroxy-4-oxoquinaldine 2,4-  **PDBTitle:** structural basis for recognition and ring-cleavage of the pseudomonas2 quinolone signal (pqs) by aqdc  **PDB Entry:** PDBe RCSB PDBj |
| 99 | c7jqxD\_ | --- \| --- \| --- | not modelled | 100.0 | 15 | **PDB header:**hydrolase **Chain:** D: **PDB Molecule:**cif-like 1 wild-type;  **PDBTitle:** crystal structure of cfl1 wild-type from burkholderia cenocepacia  **PDB Entry:** PDBe RCSB PDBj |
| 100 | c4i3fA\_ | --- \| --- \| --- | not modelled | 100.0 | 15 | **PDB header:**hydrolase **Chain:** A: **PDB Molecule:**serine hydrolase ccsp0084;  **PDBTitle:** crystal structure of serine hydrolase ccsp0084 from the polyaromatic2 hydrocarbon (pah)-degrading bacterium cycloclasticus zankles  **PDB Entry:** PDBe RCSB PDBj |
| 101 | c6cl4A\_ | --- \| --- \| --- | not modelled | 99.9 | 18 | **PDB header:**hydrolase **Chain:** A: **PDB Molecule:**lipase c12;  **PDBTitle:** lipc12 - lipase from metagenomics  **PDB Entry:** PDBe RCSB PDBj |
| 102 | c3fobA\_ | --- \| --- \| --- | not modelled | 100.0 | 16 | **PDB header:**oxidoreductase **Chain:** A: **PDB Molecule:**bromoperoxidase;  **PDBTitle:** crystal structure of bromoperoxidase from bacillus anthracis  **PDB Entry:** PDBe RCSB PDBj |
| 103 | c5hdfB\_ | --- \| --- \| --- | not modelled | 100.0 | 16 | **PDB header:**hydrolase **Chain:** B: **PDB Molecule:**hydrolase;  **PDBTitle:** hydrolase semet-stna  **PDB Entry:** PDBe RCSB PDBj |
| 104 | d2pl5a1 | --- \| --- \| --- | not modelled | 100.0 | 14 | **Fold:** alpha/beta-Hydrolases **Superfamily:** alpha/beta-Hydrolases **Family:** O-acetyltransferase **PDB entry:** PDBe RCSB PDBj |
| 105 | c5efzC\_ | --- \| --- \| --- | not modelled | 100.0 | 14 | **PDB header:**hydrolase **Chain:** C: **PDB Molecule:**homoserine o-acetyltransferase;  **PDBTitle:** monoclinic structure of the acetyl esterase mekb  **PDB Entry:** PDBe RCSB PDBj |
| 106 | c4qlaB\_ | --- \| --- \| --- | not modelled | 100.0 | 16 | **PDB header:**hydrolase **Chain:** B: **PDB Molecule:**juvenile hormone epoxide hydrolase;  **PDBTitle:** crystal structure of juvenile hormone epoxide hydrolase from the2 silkworm bombyx mori  **PDB Entry:** PDBe RCSB PDBj |
| 107 | c6qe2A\_ | --- \| --- \| --- | not modelled | 100.0 | 16 | **PDB header:**hydrolase **Chain:** A: **PDB Molecule:**monoacylglycerol lipase;  **PDBTitle:** crystal structure of paleococcus ferrophilus monoacylglycerol lipase.  **PDB Entry:** PDBe RCSB PDBj |
| 108 | c4nvrC\_ | --- \| --- \| --- | not modelled | 100.0 | 15 | **PDB header:**transferase **Chain:** C: **PDB Molecule:**putative acyltransferase;  **PDBTitle:** 2.22 angstrom resolution crystal structure of a putative2 acyltransferase from salmonella enterica  **PDB Entry:** PDBe RCSB PDBj |
| 109 | c3hjuB\_ | --- \| --- \| --- | not modelled | 100.0 | 15 | **PDB header:**hydrolase **Chain:** B: **PDB Molecule:**monoglyceride lipase;  **PDBTitle:** crystal structure of human monoglyceride lipase  **PDB Entry:** PDBe RCSB PDBj |
| 110 | c3wzlB\_ | --- \| --- \| --- | not modelled | 100.0 | 16 | **PDB header:**hydrolase **Chain:** B: **PDB Molecule:**zearalenone hydrolase;  **PDBTitle:** zen lactonase  **PDB Entry:** PDBe RCSB PDBj |
| 111 | c6azdA\_ | --- \| --- \| --- | not modelled | 100.0 | 16 | **PDB header:**hydrolase **Chain:** A: **PDB Molecule:**ppkai2-like h;  **PDBTitle:** crystal structure of physcomitrella patens kai2-like h  **PDB Entry:** PDBe RCSB PDBj |
| 112 | d1va4a\_ | --- \| --- \| --- | not modelled | 100.0 | 16 | **Fold:** alpha/beta-Hydrolases **Superfamily:** alpha/beta-Hydrolases **Family:** Haloperoxidase **PDB entry:** PDBe RCSB PDBj |
| 113 | c7e04A\_ | --- \| --- \| --- | not modelled | 100.0 | 17 | **PDB header:**hydrolase **Chain:** A: **PDB Molecule:**lipase;  **PDBTitle:** crystal structure of bomgl, a monoacylglycerol lipase from marine2 bacillus sp.  **PDB Entry:** PDBe RCSB PDBj |
| 114 | c4q3kB\_ | --- \| --- \| --- | not modelled | 99.2 | 19 | **PDB header:**hydrolase **Chain:** B: **PDB Molecule:**mgs-m1;  **PDBTitle:** crystal structure of mgs-m1, an alpha/beta hydrolase enzyme from a2 medee basin deep-sea metagenome library  **PDB Entry:** PDBe RCSB PDBj |
| 115 | c5oluA\_ | --- \| --- \| --- | not modelled | 100.0 | 15 | **PDB header:**hydrolase **Chain:** A: **PDB Molecule:**alpha/beta hydrolase family protein;  **PDBTitle:** the crystal structure of a highly thermostable carboxyl esterase from2 bacillus coagulans in complex with glycerol  **PDB Entry:** PDBe RCSB PDBj |
| 116 | c5jd6A\_ | --- \| --- \| --- | not modelled | 100.0 | 19 | **PDB header:**hydrolase **Chain:** A: **PDB Molecule:**mgs-mche2;  **PDBTitle:** crystal structure of mgs-mche2, an alpha/beta hydrolase enzyme from2 the metagenome of sediments from the lagoon of mar chica, morocco  **PDB Entry:** PDBe RCSB PDBj |
| 117 | c4f0jA\_ | --- \| --- \| --- | not modelled | 100.0 | 14 | **PDB header:**hydrolase **Chain:** A: **PDB Molecule:**probable hydrolytic enzyme;  **PDBTitle:** crystal structure of a probable hydrolytic enzyme (pa3053) from2 pseudomonas aeruginosa pao1 at 1.50 a resolution  **PDB Entry:** PDBe RCSB PDBj |
| 118 | d1imja\_ | --- \| --- \| --- | not modelled | 100.0 | 21 | **Fold:** alpha/beta-Hydrolases **Superfamily:** alpha/beta-Hydrolases **Family:** Ccg1/TafII250-interacting factor B (Cib) **PDB entry:** PDBe RCSB PDBj |
| 119 | c7a6gA\_ | --- \| --- \| --- | not modelled | 100.0 | 15 | **PDB header:**hydrolase **Chain:** A: **PDB Molecule:**putative proline iminopeptidase;  **PDBTitle:** structural characterization of l-proline amide hydrolase from2 pseudomonas syringae  **PDB Entry:** PDBe RCSB PDBj |
| 120 | c6avyA\_ | --- \| --- \| --- | not modelled | 99.9 | 18 | **PDB header:**hydrolase **Chain:** A: **PDB Molecule:**acyl-protein thioesterase 2;  **PDBTitle:** crystal structure of zea mays acyl-protein thioesterase 2  **PDB Entry:** PDBe RCSB PDBj |
